# Luigi Bogliolo: master of a glorious lineage

**DOI:** 10.4322/acr.2020.234

**Published:** 2020-11-20

**Authors:** Luiz Otávio Savassi Rocha

**Affiliations:** 1 Universidade Federal de Minas Gerais (UFMG), Faculdade de Medicina, Belo Horizonte, MG, Brasil

**Keywords:** History of Medicine, Pathology, Autopsy

## LUIGI BOGLIOLO (1908-1981)


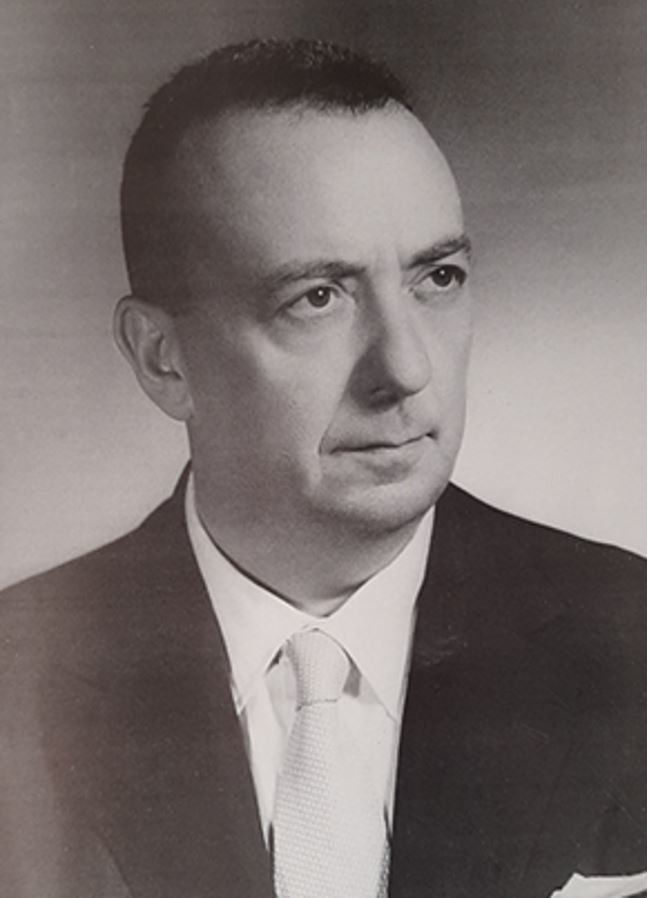


Of Italian origin but Brazilian by adoption, Luigi Bogliolo was born on April 18, 1908, on the island of Sardinia, in Sassari, the main commune of the province of the same name. He died in Belo Horizonte on September 6, 1981. The firstborn child of Enrico Bogliolo, a railroad worker, and Maria Ruju, he graduated with a medical degree from the University of Sassari in 1930 as the best student in his class. At Medical School, he was a teaching assistant in Pathological Anatomy under the guidance of Prof. Enrico Emilio Franco, who was a source of inspiration for him throughout his life. In the 1931-1932 biennium, he was a volunteer assistant at the Institute of Pathological Anatomy and Histology of the University of Sassari, directed by Franco. At the end of 1932, he moved, along with his mentor, to the Royal Adriatic University Benito Mussolini, based in Bari, where he became a staff assistant in the Pathological Anatomy Department. In December 1936, following the footsteps of Franco once more, he moved to the University of Pisa, where he remained, until March 1939, as a deputy director of the Institute of Pathological Anatomy and Histology.

An Italian of Jewish origin, Enrico Emilio Franco (*Salomone* Enrico Franco) was born in Trieste on November 22, 1881, and graduated with a degree in medicine from the Royal University of Padua in 1906. He was very demanding as a teacher, and apparently his students feared him with the same intensity with which they admired him; nonetheless, in the case of Bogliolo, he created all the conditions for the emergence of the “master” that his disciple, perhaps unsuspectingly, held in the core of his own being. His classes on the cadaver in the autopsy room were *belissime*, as Prof. Giuseppe Sangiorgi stated in the lecture (posthumous eulogy) that he delivered in Bari on November 18, 1950, to the members of the Italian Society for Experimental Biology. On that topic, his manual atlas of autopsy techniques, originally published in 1926, is an indisputable testimony of the seriousness and mastery with which he performed cadaveric examinations, which he considered a thorough investigation and not a hasty exercise in organ extraction.^1^ Thus, he did not approve of having clocks in the autopsy room since the procedure had a specific start time but no specific end time.

While they were working in Pisa, Bogliolo and Franco bonded affectionately and professionally with the regent of the Chair of General Pathology, Cesare Sacerdotti, a disciple of Camillo Golgi and Giulio Bizzozero, whom they respectfully referred to as *Il Professore*. In a demonstration of the prestige that they enjoyed in the Pisan university circles, the three friends were known as *I tre sofi* (“The three wise men”), not only because of their high intellectual stature but also because they descended from a glorious scientific lineage, which is summarized below.

As mentioned, Luigi Bogliolo (1908-1981) was a direct disciple of Enrico E. Franco (1881-1950) who, as a medical graduate from the University of Padua in 1906, was linked to the School of Giovanni Battista Morgagni (1682-1771), a distinguished professor of that old university and the author of the masterpiece **De sedibus et causis morborum per anatomen indagatis** – a systematic confrontation between autopsy findings and clinical records, published in 1761. Therefore, one can consider Enrico E. Franco a legitimate descendant of the “Father of Pathological Anatomy”, from whom he was distanced by a few generations.

Giovanni Battista Morgagni was a disciple of the anatomist and surgeon Antonio Maria Valsalva (1666-1723), a professor at the University of Bologna who dedicated himself to the study of the anatomy, physiology and pathology of the human ear. Valsalva was a disciple of Marcello Malpighi (1628-1694), founder of microscopic anatomy and author of notable contributions such as the first observation of capillary circulation, which was previously inferred by William Harvey; the first description of the vesicular structure of the human lung, which formed the basis for the formulation of a theory of respiration; the description of the lymphatic follicles of the spleen; the pioneering description of renal glomeruli; a detailed description of the structure and metamorphosis of the silkworm and of plant morphology; the description of the prickle cell layer of the skin; and the establishment of the foundations of a new discipline, Embryology. Marcello Malpighi was, in turn, both a mentor and a disciple of the physicist and mathematician Giovanni A. Borelli (1608-1679), the founder of Iatromechanics (the application of mechanics to medicine). Finally, Borelli was a disciple of the physicist and mathematician Galileo Galilei (1564-1642), a pioneer of scientific thought.

The connections between Bogliolo and Franco with Cesare Sacerdotti allow the exploration of a second branch of what could be called the scientific lineage of Bogliolo. Sacerdotti was a direct disciple of Camillo Golgi (1843/44-1926), who, along with Santiago Ramón y Cajal, won, in 1906, the Nobel Prize in Physiology or Medicine for his brilliant studies on the fine structure of the nervous system; he was also a disciple and collaborator of Giulio Bizzozero (1846-1901), the discoverer of platelets (and of their role in hemostasis) and of spiral bacteria in the stomach of dogs, the first step towards the identification of the species *Helicobacter pylori* in the human stomach. Giulio Bizzozero studied in Germany with Rudolf Virchow (1821-1902), author of the magnum opus **Die Cellularpathologie** (1858) and of the famous aphorism *Omnis cellula e cellula,* which is why he is nicknamed the “Father of Cellular Pathology.” In addition, Camillo Golgi himself was a disciple of Giulio Bizzozero.

**Figure 1:**
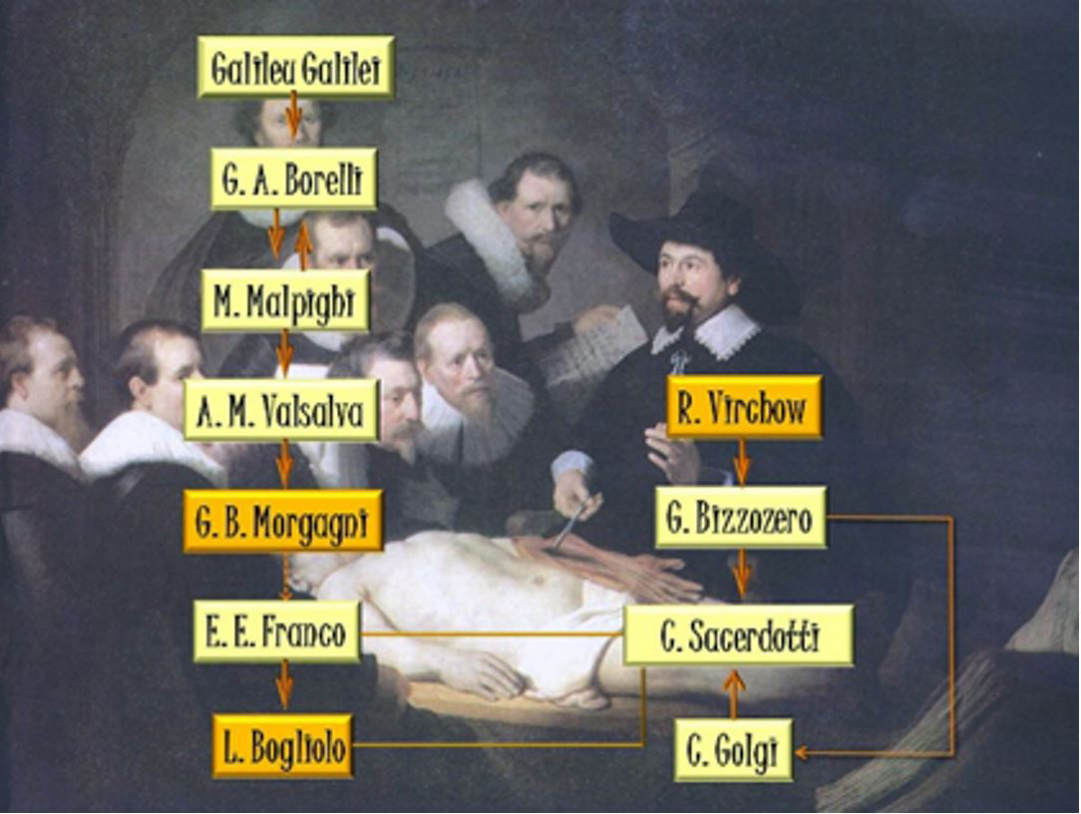
Diagram of the scientific lineage of Luigi Bogliolo. In the background, the famous painting by Rembrandt van Rijn, “The anatomy lesson of Dr. Tulp”, dated 1632.

Figure 1 shows the connections of Bogliolo with the School of Morgagni (the “Father of Pathological Anatomy”) and with the School of Virchow (the “Father of Cellular Pathology”), illustrating a glorious scientific lineage to which anyone would be proud to belong.

The publications of Bogliolo during his academic life in Italy deal with various topics, with an emphasis on those related to leishmaniasis and, in particular, to the experimental production of sarcomas through injections of Thorotrast in the mouse (*Mus musculus*, var. *alba*) and the rat (*Mus norvegicus*).

Thorotrast (a radiological contrast based on radioactive thorium dioxide, whose biological half-life in humans is 200-400 years) was introduced for clinical use in 1928 and was used initially for retrograde pyelography and intravenous hepatolienography. As expected, almost all of the injected thorium dioxide was retained in the body, with 70% deposited in the liver, 20% in the spleen and the remainder in the reticuloendothelial system of the bone marrow and in the periportal lymph nodes. In 1931, Thorotrast began to be used by the Portuguese neurosurgeon Egas Moniz to examine the intracranial vessels, replacing the iodinated contrast that had been used until then for the same purpose.^2^ Despite the quality of the radiographs obtained, accidental extravascular injection of the contrast in the groin, cubital fossa and neck resulted in severe tissue reactions (*thorotrastomas*) due to exposure to alpha radiation (intense fibrosis, with the formation of hardened masses that caused functional limitation of the affected areas and compressed the adjacent structures).

**Figure 2:**
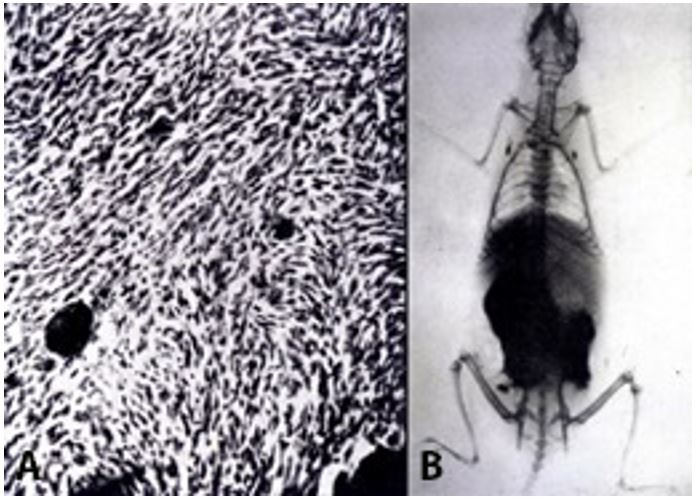
**A –** Fasciculated spindle cell sarcoma produced by Luigi Bogliolo via the injection of Thorotrast in a mouse (*Mus musculus* var. *alba*). Globules and granules of thorium dioxide are shown inside the tumor; **B –** Radiography of a rat (*Mus norvegicus*) that received fractional doses of the radiological contrast between October 19, 1936, and February 7, 1937, in the subcutaneous tissue of the caudal half of the abdomen. Notably, 78 days after the end of treatment, most of the radiopaque substance (radioactive thorium dioxide) remains *in situ* but is also found in the axillary, mediastinal and inguinal lymph nodes.

In 1937, Luigi Bogliolo demonstrated the development of fasciculated spindle cell sarcoma in mice *(Mus musculus*, var. *alba*) with successive injections of Thorotrast subcutaneously into the posterior half of the abdomen. In a second study, published in 1938, the injection of the contrast in the rat (*Mus norvegicus*) was able to induce the appearance of malignant blastomas in all 23 animals in the experiment, including 20 cases of fasciculated spindle cell sarcoma and three cases of pleomorphic sarcoma with areas of cartilaginous metaplasia (Figure 2).^3^ ^,^ ^4^

A late complication of the indiscriminate use of Thorotrast in clinical practice was the appearance, first observed in 1947, of malignant neoplasms (especially cholangiocarcinomas and hepatic hemangioendotheliosarcomas) in hundreds of patients who had received the radiological contrast for diagnostic purposes, sometimes as much as 40 years previously. Nonetheless, the use of Thorotrast continued until the mid-1950s at highly prestigious institutions such as the Massachusetts General Hospital, the Lahey Clinic and the University of Michigan Hospital, in one of the most embarrassing episodes in the history of medicine.

In Pisa, Bogliolo obtained the title of full professor in a public examination held in 1938; that same year, he married Geula Bennoun, a medical student of Jewish origin born in Jaffa, a twin city of Tel Aviv. In January 1939, Franco was dismissed from the University of Pisa amid the wave of anti-Semitism that ravaged his homeland and was forced into exile in Jerusalem, where he died on September 20, 1950, from diabetic nephropathy. In March 1939, Bogliolo was dismissed from the same University for his antifascist position, aggravated by his close relationship with Professor Franco and with Geula Bennoun, and was forced to leave his native country as well. After spending a few months in Belgium, he traveled with his wife to Brazil, arriving in Rio de Janeiro on January 5, 1940. In exile, until Franco's death, master and disciple never ceased to correspond, despite the difficulties imposed by World War II, which began on September 1, 1939, and the Arab-Jewish conflict, which experienced its most dramatic moments in 1947-1948. In January 1949, Bogliolo and Franco met, for the last time, in Jerusalem.

At the beginning of his life in Rio de Janeiro, Luigi Bogliolo and his wife lived in a second-class guest house near the pier; and to survive, they tanned alligator skins in the bedroom. It is known that ancient Jews knew the method of tanning hides and skins with oak bark, an art that they must have learned as captives in Egypt; however, in the case of the young couple newly arrived from Italy, the place – a small bedroom in a guest house – was not the most suitable for the exercise of that ancient practice. It did not take long for problems to arise: annoyed by the strong smell from the tanning process, the owner of the guest house used to pound the door of the bedroom asking for explanations and threatening to evict the two afflicted artisans who, in an attempt to hide their wrongdoing, quickly threw the skins under the bed.

In January 1941, under the recommendation of Prof. Eduardo MacClure, Bogliolo took on the Pathological Anatomy Service of the 5th Chair of Internal Medicine of the National Faculty of Medicine (now Faculty of Medicine of UFRJ), led by the great clinician Heitor Annes Dias (1884-1943), nicknamed “the Brazilian Jiménez Díaz”. In perfect harmony with Annes Dias, Bogliolo boosted the Clinicopathological Conferences at that institution. Inspired by the unforgettable lessons of Franco, he conducted the autopsies of the cases that were presented in those meetings. Thus, after the clinical features had been explored, the diagnostic hypotheses had been formulated, and the possible reasons for the death of the patient had been considered, he was in charge of the autopsy report. Furthermore, at the end of the conferences, he used to perform the great synthesis – the epicrisis – correlating the *post mortem* findings with those observed *intra vitam*.

With the premature death of Annes Dias in 1943, Bogliolo was invited by his compatriot Alfredo Balena, director of the Faculty of Medicine of Belo Horizonte (now Faculty of Medicine of UFMG), to head the Chair of Pathological Anatomy of the institution Balena had helped to found in 1911. Starting in 1944, Bogliolo continued to perform autopsies and to favor Clinicopathological Conferences in his new home until his compulsory retirement in 1978. Certainly, he heeded Morgagni’s wise warning that the physicians who have done or seen many autopsies have learned at least to mistrust their diagnoses, while those who do not confront themselves with the often discouraging findings of *post mortem* examination, live in the clouds of a vain illusion. Forty-two years after Bogliolo’s retirement, the Clinicopathological Conferences continue to take place at the Faculty of Medicine of UFMG. Regarding the invaluable pedagogical value of this academic activity, which I have coordinated since 1995, I published a book in 2010^5^ and wrote an editorial in 2013.^6^

In the autopsy room, which he considered a kind of “sacred temple”, Bogliolo demanded absolute respect for the corpse, as if he incorporated the message contained in an ancient Latin maxim – *Taceant colloquia. Effugiat risus. Hic locus est ubi mors gaudet succurrěre vitae* – according to which, in the place where death delights to help the living, there should be no talking or laughing. To describe the Bogliolo’s austere demeanor during the performance of an autopsy, it is worth reproducing the testimony of the late psychiatrist Joaquim Affonso Moretzsohn, who graduated in 1946:^7^

*I met Prof. Bogliolo. He debuted with my class in the 4th year of Medical School, in 1944. ‘What? Do you want to smell roses?’ he exclaimed with a heavy peninsular accent when he realized that I took a handkerchief to my nose in the autopsy room.*

As mentioned, in 1944, Luigi Bogliolo became the head of the Chair of Pathological Anatomy at the Faculty of Medicine of Belo Horizonte. In the capital of Minas Gerais State, three of his children (Ada, Rina and Anna Rosa) were born, whereas Alexandre, his only son, had already been born in Rio de Janeiro in 1942. Both Alexandre and Ada would tragically die very young, victims of accidents, respectively in 1965 and 1967. Because he was a foreigner, Bogliolo was forced to revalidate his diploma; to accomplish this goal, between 1953 and 1957, he underwent practical, written and oral exams for all subjects of the last three years of the medical program. Therefore, it was not until 1958 that he completed his doctorate, defending a thesis on the pathology of acute toxemic form of schistosomiasis mansoni. In 1959, after a brilliant examination that consisted of an analysis of his *curriculum vitae,* a written exam, a practical exam, a didactic exam and a thesis defense, he achieved the Cathedra.

In 1972, through the Editora Guanabara Koogan, Bogliolo published the first edition of the book **Patologia**, with the participation of his assistants and renowned professors throughout Brazil and Italy. In 2016, the ninth edition of the book, with 1,542 pages, was printed under the supervision of Prof. Geraldo Brasileiro Filho. In 1978, also through the Editora Guanabara Koogan, Bogliolo published the book **Patologia Geral Básica**; in 2018, also under the supervision of Prof. Geraldo Brasileiro Filho, the sixth edition of the book was printed.

Working at the Faculty of Medicine of UFMG until 1978, the year of his compulsory retirement, Bogliolo created a School of Pathological Anatomy that through its high level of regard would impose itself onto the scientific environment of his adoptive country. His first assistant professors included Paulo Roberto Ferreira Borges, Nello de Moura Rangel, Iracema Baccarini, João Henriques de Freitas Filho, Edmundo Chapadeiro, Washington Luiz Tafuri and Pedro Raso. The contributions of his School cover various topics in the field of Pathology, with an emphasis on tropical diseases, such as schistosomiasis mansoni and Chagas disease. The contributions of Bogliolo himself to the knowledge of the pathology of the different anatomoclinical forms of schistosomiasis mansoni deserve special attention, and include the following:

• the accurate characterization of the morphological findings of the liver in the hepatosplenic form, especially in the case of Symmers fibrosis, which, according to some authors, should be called “Symmers-Bogliolo fibrosis”;

• the clarification of the concepts of fibrosis and cirrhosis as they apply to fibrosing liver diseases, thus distinguishing the anatomical picture of the liver in the hepatosplenic form of schistosomiasis and in visceral leishmaniasis from that of liver cirrhosis;

• the first description, in patients with the hepatosplenic form undergoing splenoportography, of the peculiar changes in the hepatogram, characterized by the presence, next to the clearer and denser image of the dichotomous portal branches, of a less dense and less defined shadow attributed to the newly formed vascular network around the dichotomous branches (“Bogliolo’s sign”);

• the detailed description of the anatomical picture of the acute toxemic form, which was the focus of his doctoral thesis.

The post-graduation, so valued today, was, in a way, already being carried out by Bogliolo in the 1950s; in fact, it consisted of a “post-graduation in spirit”, because it lacked regulation and recognition by the relevant authorities. It was only in 1972 that the Teaching and Research Coordination Office of UFMG approved the creation of the Post-Graduate Program in Pathology, with three areas of concentration: General Pathology, Special Medical Pathology and Special Dental Pathology.

Luigi Bogliolo was one of the founders of the Brazilian Society of Pathologists and served in virtually all positions therein, including the presidency (biennium 1960-1962). His remarkable presence, his verve, his critical spirit and his unmistakable style were felt in the conferences promoted by that society. He was also a member of the Minas Gerais Academy of Medicine, occupying chair 46, whose patron is José Aroeira Neves, a disciple of Ezequiel Dias. In 1978, shortly after his compulsory retirement, he received the title of Professor Emeritus of the Faculty of Medicine of UFMG.

Although he was extremely strict and demanding, to the point – like Prof. E. E. Franco – of stoking undisguised fear in many of those who interacted with him, Bogliolo left an indelible mark on his disciples and collaborators. After all, one of his great passions was the unlocking of young people’s potential, encouraging them to exercise reasoning, ask relevant questions, develop critical awareness and cultivate curiosity. Thus, he certainly endorsed the words of the ancient Greek philosopher Plutarch:^8^

*For the mind does not require filling like a bottle, but rather, like a wood, it only requires kindling to create in it an impulse to think independently and an ardent desire for the truth*.

In 1992, I published a book about the fascinating trajectory of the late master.^9^ In January 2008, at the request of Prof. Marcello Franco, head of the Department of Pathology of the Paulista School of Medicine (UNIFESP), I sent a copy of the book to the respected Australian pathologist Robin Cooke, editor of the Bulletin of the International Academy of Pathology, who used the material in a conference entitled “The influence of European Pathologists throughout the World”. In that conference, when referring to Brazil, Robin Cooke highlighted three other European pathologists in addition to Luigi Bogliolo – Walter Haberfeld, Walter Büngeler and Fritz Köberle – who contributed greatly to the consolidation of the specialty in their adoptive country.
